# Ferroptotic therapy in cancer: benefits, side effects, and risks

**DOI:** 10.1186/s12943-024-01999-9

**Published:** 2024-05-03

**Authors:** Jiandong Diao, Yuanyuan Jia, Enyong Dai, Jiao Liu, Rui Kang, Daolin Tang, Leng Han, Yingjie Zhong, Lingjun Meng

**Affiliations:** 1 2nd Inpatient Area of Oncology and Hematology Department, China-Japan Union Hospital of Jilin University, Changchun, Jilin, 130031 China; 2 DAMP laboratory, The Third Affiliated Hospital of Guangzhou Medical University, Guangzhou, 510120 Guangdong China; 3 Department of Surgery, University of Texas Southwestern Medical Center, Dallas, Texas USA; 4 Department of Pediatrics, China-Japan Union Hospital of Jilin University, Changchun, Jilin, 130031 China

**Keywords:** Ferroptosis, Targeted therapy, Cancer, Benefits, Side effects

## Abstract

Ferroptosis is a type of regulated cell death characterized by iron accumulation and uncontrolled lipid peroxidation, leading to plasma membrane rupture and intracellular content release. Originally investigated as a targeted therapy for cancer cells carrying oncogenic *RAS* mutations, ferroptosis induction now exhibits potential to complement chemotherapy, immunotherapy, and radiotherapy in various cancer types. However, it can lead to side effects, including immune cell death, bone marrow impairment, liver and kidney damage, cachexia (severe weight loss and muscle wasting), and secondary tumorigenesis. In this review, we discuss the advantages and offer an overview of the diverse range of documented side effects. Furthermore, we examine the underlying mechanisms and explore potential strategies for side effect mitigation.

## Introduction

Cell death is a fundamental biological process inherent in complex organisms, serving as a critical mechanism for the elimination of dysfunctional or aging cells [1]. This process comprises two primary categories: accidental cell death, occurring in response to unexpected insults and injuries, and regulated cell death, a finely tuned machinery susceptible to modulation by drugs or genetic interventions [2]. Regulated cell death plays a pivotal role not only in tissue development and cellular homeostasis, but also contributes to various diseases and pathological conditions, including cancer, neurodegeneration, infections, and ischemia-reperfusion damage. In the context of cancer treatment, the induction of regulated cell death has become a primary goal in various targeted therapies, enabling the precise eradication of tumor cells while minimizing harm to healthy cells [3].

**Fig. 1:**
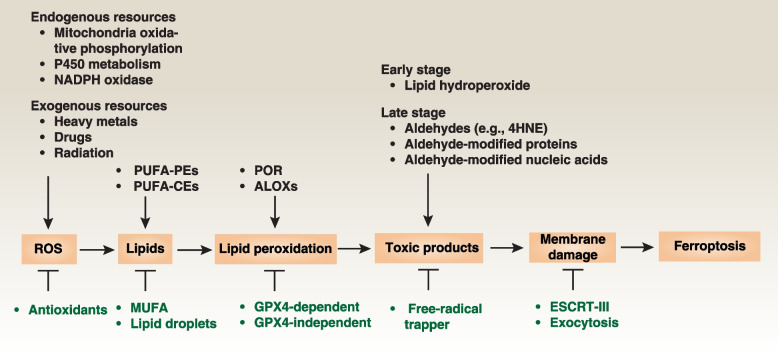
A summary of the process of ferroptosis. Ferroptosis is a form of regulated necrosis primarily driven by lipid peroxidation, resulting in membrane damage and rupture. Increased production of ROS (reactive oxygen species) from endogenous or exogenous sources can initiate lipid peroxidation of PUFA (polyunsaturated fatty acids), which are a major component of cell membranes. The toxic byproducts of lipid peroxidation, including early-stage lipid hydroperoxides and late-stage aldehydes, as well as aldehyde-modified molecules, impair membrane structure and function. In contrast, various defense systems are capable of blocking or delaying this oxidative damage

Considering the limitations associated with current drug treatments, often leading to apoptosis resistance, the exploration of non-apoptotic cell death has arisen as a logical strategy in both basic research and clinical trials. Specifically, the term 'ferroptosis,' coined in 2012, delineates a distinctive form of non-apoptotic cell demise marked by uncontrolled lipid peroxidation [4] (Fig. 1). Traditional cell death effectors, such as caspases, GSDMD (gasdermin D), and MLKL (mixed lineage kinase domain like pseudokinase), are not essential for the process of ferroptosis [4]. Ferroptosis can be categorized as a type of regulated necrosis and exhibits some morphological characteristics reminiscent of necrotic cell features, such as plasma membrane rupture [5]. Mechanistically, ferroptosis is mediated by the generation of toxic oxidized lipids, including 4-hydroxynonenal [6], as a result of lipid peroxidation [7]. Additionally, advanced lipid peroxidation end products can lead to oxidative damage to proteins or nucleic acids, causing cellular dysfunction [8]. Conversely, various antioxidant systems, comprising both GPX4 (glutathione peroxidase 4)-dependent and GPX4-independent pathways, play a context-dependent role in defending against ferroptosis [9–18].

In recent years, there has been growing interest in using ferroptosis activators as a cancer treatment approach [19]. Tumor cells with metastatic potential or resistance to apoptosis-based therapies have demonstrated susceptibility to ferroptosis. Various interventions, including experimental compounds and clinically used drugs (Table 1), have shown promise in inducing ferroptosis for cancer therapy, even overcoming resistance to traditional treatments [20]. Furthermore, combination therapies involving ferroptosis induction hold potential to enhance the efficacy of conventional treatments (such as radiotherapy and immunotherapy), while preventing tumor recurrence [19, 21]. However, the current ferroptosis induction strategy can lead to significant side effects, highlighting the need for a deeper understanding of its role in cancer therapy [22].

**Table 1:** Key ferroptosis inducers in tumor therapy

**Name**	**Mechanisms**	**IC50**	**Models**	**Note**	**References**
**Experimental compounds**					
Erastin	Inhibits system xc^-^	1.2 μM in HT1080	Various cancer cell lines, especially BJ-TERT/LT/ST/RAS^V12^, HT1080 and Calu-1		[4]
Erastin2	Inhibits system xc^-^	0.15 µM in HT1080	HT1080 and Calu-1		[23]
Imidazole ketone erastin (IKE)	Inhibits system xc^-^	3 nM in BJ-TERT/LT/ST/RAS^V12^	BJ-TERT/LT/ST/RAS^V12^, HT1080 and Calu-1; xenograft model; orthotopic model	Metabolically stable inhibitor of system xc^-^, potentially suitable for in vivo applications	[24]
L-Buthionine-(S,R)-Sulfoximine (BSO)	Inhibits GCLC	26.5 µM in MCF-7	Various cancer cell lines, especially HT1080 and Calu-1	BSO alone may not be sufficient to induce ferroptosis in certain cancers	[4, 25]
RSL3	Inhibits GPX4	100 nM in Pfa1	Various cancer cell lines, especially HT1080 and Calu-1; xenograft model	It has off-target effects on TXNRD1	[4, 26]
ML162	Inhibits GPX4	25 nM in BJ-TERT/LT/ST/RAS^V12^	Various cancer cell lines, especially BJ-TERT/LT/ST/RAS^V12^, HT1080 and Calu-1	It has off-target effects on TXNRD1	[26–28]
ML210	Inhibits GPX4	71 nM in BJ-TERT/LT/ST/RAS^V12^	Various cancer cell lines, especially BJ-TERT/LT/ST/RAS^V12^, HT1080 and Calu-1		[27]
JKE-1674	Inhibits GPX4	0.03 µM in LOX-IMVI	Various cancer cell lines, especially HT1080 and LOX-IMVI	Active metabolite of ML210	[27]
JKE-1716	Inhibits GPX4	N/A	Various cancer cell lines, especially HT1080 and LOX-IMVI	Derivative of ML210	[27]
FIN56	Inhibits GPX4	5 µM in HT1080	Various cancer cell lines, especially HT1080	It also binds to and activates FDFT1/SQS	[29]
FINO2	Inhibits GPX4	5.8 µM in HT1080	Various cancer cell lines, especially HT1080	Oxidizes iron, resulting in the loss of GPX4 enzymatic activity	[30]
iFSP1	Inhibits AIFM2/iFSP1	3 µM in Pfa1	GPX4-knockout Pfa1 and HT1080 cells that overexpresses AIFM2	iFSP1 alone may not be sufficient to induce ferroptosis in certain cancers	[12]
icFSP1	Inhibits AIFM2/iFSP1	30 µM in HT1080	Human cancer cells (H460, A375, and HT1080 cells); xenograft model	It indirectly inhibits AIFM2/FSP1 by inducing condensate formation	[14]
Torin 1	Inhibits MTOR	10 nM (cell free system)	UMRC6		[31]
ZZW-115	Inhibits NUPR1	2.1 µM in PANC1	PANC1, MiaPaCa-2 and HepG2		[32, 33]
Alkaloid trigonelline	Inhibits NFE2L2	N/A	HepG2, Hepa1–6, Hep3B, and SNU-182; xenograft model		[34]
Brequinar	Inhibits DHODH	20 nM (cell free system)	Various cancer cell lines, especially NCI-H226 and HT1080cells	It has off-target effects on AIFM2	[13]
MF-438	Inhibits SCD/SCD1	2.3 nM (cell free system)	KYSE30, KYSE70, KYSE140, KYSE150, KYSE410, KYSE450, KYSE510 and SHEE cells		[35]
N6F11	Activates TRIM25	5 µM in PANC1	Various cancer cell lines, especially PANC1	Induces TRIM25-dependent GPX4 degradation in cancer cells	[36]
**Clinical drugs**					
Sulfasalazine	Inhibits system xc^-^	5 mM in HT1080	Various cancer cell lines, especially HT1080 and Calu-1	An anti-inflammatory drug structurally related to salicylates. It is indicated for managing inflammatory diseases such as ulcerative colitis and rheumatoid arthritis	[37]
Sorafenib	Inhibits SLC7A11	4.5 µM in HepG2	Various cancer cell lines, especially HT1080 and HepG2; xenograft model	A kinase inhibitor used to treat unresectable liver carcinoma, advanced renal carcinoma, and differentiated thyroid carcinoma; Sorafenib may not trigger ferroptosis in certain cancer types	[37, 38]
Statins (lovastatin, simvastatin)	Inhibit HMG-CoA reductase	Lovastatin: 1.4 nM simvastatin: 0.12 nM (cell free system)	HT1080 and Calu-1	A class of lipid-lowering medications	[29, 39]
Artemisinin	Increases iron accumulation	20.36 nM (cell free system)	Various cancer cell lines, especially NCI-H292 and HCT116	It is used to treat malaria	[40]
Artesunate	Increases iron accumulation	1.28 nM (cell free system)	Various cancer cell lines, especially HT1080 and Calu-1	It is used to treat malaria	[41] [42]
Rapamycin	Inhibits MTOR	0.5 ng/ml in rhabdomyosarcoma cells	PANC1	An immunosuppressive agent	[43]
Abemaciclib	Inhibits CDK4/6	10 nM (cell free system)	ER+ breast cancer cell lines; xenograft model	It is used to treat is used to treat certain types of breast cancer known as HR^+^/HER2^–^	[44]
Disulfiram	Induces mitochondrial damage	0.1 mM (cell free system)	GBM U251 and LN229	It is used to treat chronic alcoholism	[45]
Doxorubicin	Increases PUFA	40 µM (cell free system)	Cardiomyocytes	They are chemotherapy medicines used to treat certain types of cancer	[46]
Oxaliplatin	Induces ROS production	2.7 µM in KB-CP20	HT29 and KB-CP20	An alkylating agent used to treat certain types of cancer	[47]
Cisplatin	Induces GSH depletion	2 µM in HOS	A549, HOS and HCT116	An alkylating agent used to treat certain types of cancer	[48]
Lapatinib	Increases iron accumulation	10.8 nM (cell free system)	MDA MB-231	A tyrosine kinase inhibitor used for the treatment of advanced or metastatic HER2^+^ breast cancer	[49]
Neratinib	Increases iron accumulation	92 nM (cell free system)	TBCP-1 and SKBR3; xenograft model	A tyrosine kinase inhibitor used for the treatment of advanced or metastatic HER2^+^ breast cancer	[49]
Zalcitabine	Induces mitochondrial DNA stress	20 µM in PANC1	PANC1 and Capan2; xenograft model	A dideoxynucleoside antiretroviral agent used for the treatment of human immunodeficiency virus	[50]

In this review, we discuss the benefits and risks of ferroptosis-based antitumor therapy. Our aim in this introduction is to provide insights into strategies for mitigating these limitations and optimizing ferroptosis-based cancer treatments.

### The discovery of ferroptosis

Erastin and RSL3 are two well-known small molecule compounds that are frequently used to trigger ferroptosis and explore the associated mechanisms of this cell death pathway. However, the discovery and utilization of erastin and RSL3 preceded the formal recognition of the term 'ferroptosis'.

In 2003, the laboratory of Brent Stockwell utilized synthetic lethal high-throughput screening, assessing a vast library of 23,550 compounds for their ability to selectively eliminate engineered tumorigenic cells (BJ-TERT/LT/ST/RAS^V12^) while sparing their isogenic normal cell counterparts [51]. Through this screening, they identified erastin as a novel compound capable of selectively killing engineered tumorigenic cells with *RAS* mutations, but not wild type cells. This cell death induced by erastin was found to be caspase-independent [51]. In 2008, the same research group conducted a similar screening involving 47,725 compounds to target BJ-TERT/LT/ST/RAS^V12^ cells [52]. This effort led to the discovery of RSL3, another compound that selectively kills *RAS*-mutant cells through a caspase-independent mechanism. In contrast to erastin, RSL3 activates a similar death mechanism, but in a mitochondrial VDAC (voltage-dependent anion channel)-independent manner.

Subsequent studies have shown that erastin-induced cell death in the fibrosarcoma cell line HT1080 and the lung cancer cell line Calu-1 relies on iron accumulation, consequent oxidative damage through the activation of the Fenton reaction, and inhibition of system xc^-^ [4]. System xc^-^ is an amino acid antiporter that facilitates the exchange of extracellular cystine and intracellular glutamate across the cellular plasma membrane. It comprises a heavy chain component, SLC3A2 (solute carrier family 3 member 2), and a transport module, SLC7A11 (solute carrier family 7 member 11) [53]. The term 'ferroptosis' was introduced in 2012 to describe this iron-dependent, non-apoptotic form of cell death. Subsequent research has demonstrated that RSL3 directly binds to and inhibits GPX4, a downstream molecule in the cystine uptake and subsequent GSH (glutathione) synthesis pathway mediated by the system xc^-^, following the reduction of cystine to cysteine [9]. The current list of GPX4 inhibitors is expanding, with ML210 standing out as a covalent GPX4 inhibitor, utilizing intracellular drug metabolism to target the selenocysteine residue of GPX4 [27]. Nevertheless, ongoing debates persist regarding whether RSL3 and ML210 should selectively target GPX4, rather than other proteins in this context [26]. While GSH is the preferred co-factor for GPX4, it can also utilize other thiol-containing proteins as reductants [54]. In general, classical ferroptosis is triggered by erastin and RSL3, both of which inhibit the system xc^-^-GSH-GPX4 pathway. This inhibition results in the accumulation of ROS (reactive oxygen species) within cells, ultimately leading to unrestricted lipid peroxidation.

It's worth mentioning that the early mechanisms of ferroptosis exhibit similarities to oxytosis, a form of cell death observed in neuronal cells, including the HT-22 cell line [55]. Oxytosis involves glutamate-induced inhibition of system xc^-^, subsequent depletion of GSH, production of ROS, and activation of lipoxygenases and heat shock proteins [56]. The depletion of cystine or cysteine in the cell culture medium can also trigger ferroptotic cell death [57]. However, our current understanding of ferroptosis extends beyond the oxytotic phenotype, as we will discuss later, due to the discovery of GPX4-independent ferroptotic pathways. Furthermore, the induction of ferroptosis is not limited to cells with *RAS* mutations but has been observed in normal tissues or *RAS* wild-type cells [58, 59], highlighting the context-dependent nature of ferroptosis.

### Positive regulators of ferroptosis

Given the intricate connection between the ferroptotic process and oxidative stress-induced lipid peroxidation, the synergistic interplay of ROS amplification, lipid provisioning, and activation of lipid peroxidation enzymes collectively contribute to fostering ferroptosis induction or augmenting ferroptosis sensitivity (Fig. 1).

### ROS generation

ROS are chemically reactive molecules containing oxygen, typically produced as natural byproducts of cellular metabolism. They are essential for various physiological processes within cells. However, excessive accumulation of ROS can lead to oxidative stress, causing damage to cellular components (e.g., DNA, proteins, and lipids), and potentially contributing to various diseases. There are three main sources that generates ROS for ferroptosis:

- 1) Mitochondria: Mitochondria serve as the primary source of ROS during oxidative phosphorylation, a process vital for cellular energy generation. Electrons escaping from the electron transport chain can react with molecular oxygen, leading to the production of O_2_^·−^ (superoxide radicals), a type of ROS. Mitochondrial ROS can act as triggers for ferroptosis, while the presence of mitochondrial antioxidant systems, comprised of enzymatic and non-enzymatic antioxidants, can mitigate ferroptosis [50, 57, 60–66]. Multiple mitochondrial metabolic pathways influence ATP (adenosine triphosphate) and ROS generation, thereby influencing ferroptosis sensitivity. For instance, glutaminolysis assumes particular importance in scenarios with limited glucose availability, such as in rapidly growing tumors. Cancer cells often depend on glutaminolysis to fulfill their high energy and biosynthetic demands. However, glutaminolysis can promote ferroptotic death triggered by deprivation of full amino acids or of cystine alone [57, 67]. The AMPK (AMP-activated protein kinase) functions as a critical cellular energy sensor. When activated in response to declining energy levels, AMPK promotes ATP production by enhancing the activity or expression of catabolic proteins. Simultaneously, it conserves ATP by inhibiting biosynthetic pathways. AMPK activation during energy deficiency can both inhibit ferroptosis, through phosphorylation of ACACA/ACC (acetyl-CoA carboxylase alpha) in MEFs (mouse embryonic fibroblasts) and human renal adenocarcinoma cells [68], and promote ferroptosis, by targeting BECN1 (beclin1)-mediated system xc^-^ inhibition in human colorectal cancer cells [25] or regulating pyrimidinosome assembly in human cervical cancer cells [69]. These findings suggest the presence of a threshold for AMPK activity in regulating ferroptosis levels in cancer cells through various substrates or binding proteins.
- 2) NOXs (NADPH oxidases): These enzymes are specialized proteins that generate ROS as part of their normal function. While they serve crucial roles in immune responses and cellular signaling, they also contribute to ferroptosis. Increased expression of NOX can result in elevated ROS levels, thereby increasing sensitivity to ferroptosis [70–72]. The activity of NOX in ferroptosis is subject to regulation by various factors. For example, the tumor suppressor protein TP53/p53 (tumor protein p53) can inhibit ferroptosis in human colorectal cancer cells by binding to DPP4 (dipeptidyl peptidase 4) [70]. Conversely, TP53 deficiency promotes the accumulation of DPP4 on the cell membrane, where it forms a complex with NOX1, resulting in oxidative damage [70]. Arachidonic acid also has the capacity to enhance NOX1 activity through phosphorylation by PRKC/PKC (protein kinase C), thereby promoting ROS production [73]. Furthermore, 4-hydroxynonenal, a byproduct of lipid peroxidation, augments NOX1 activity and induces ferroptosis in HT1080 and Calu1 cells [6]. The ferroptosis-inducing effect of NOX1 activity can be counteracted by ALDH1B1 (aldehyde dehydrogenase 1 family member B1) in HT1080 and Calu-1 cells [6]. ALDH1B1 catalyzes the oxidation of aldehydes, converting them into their corresponding carboxylic acids, a process relevant to the development of colorectal and pancreatic tumors [74, 75].
- 3) The fenton reaction. The fenton reaction is a chemical process that occurs when H_2_O_2_ (hydrogen peroxide) interacts with a metal catalyst, usually Fe^2+^, resulting in the formation of highly reactive and destructive ·OH (hydroxyl radicals) [76]. This reaction is a notable contributor to oxidative stress and can cause damage to cellular components. Hydroxyl radicals target and harm the lipids found in cell membranes, namely lipid peroxidation. Lipid peroxidation can result in membrane destabilization, compromising the integrity of cells and potentially leading to ferroptosis. Apart from its involvement in the fenton reaction, iron can also enhance the activity of enzymes, such as ALOX (arachidonate lipoxygenase) family and POR (cytochrome p450 oxidoreductase), thereby increasing ferroptosis sensitivity. Consequently, alterations in iron metabolism, including processes, such as iron uptake, storage, utilization, and release, can modulate ferroptosis sensitivity. An extensively studied example is the induction of ferritinophagy, a selective form of autophagy that promotes the degradation of the iron storage protein ferritin in MEFs or pancreatic cancer cells, resulting in an increase in labile iron [77, 78]. This process has been demonstrated to enhance ferroptosis sensitivity in various disease models [79–83].

### PUFA synthesis

PUFAs (polyunsaturated fatty acids) are a category of dietary fats characterized by having two or more double bonds within their molecular structure. These double bonds introduce kinks into the fatty acid chains, preventing them from closely packing together. Consequently, PUFAs typically maintain a liquid state at room temperature and are commonly referred to as "healthy" or "good" fats. PUFAs are essential for the human body and serve critical roles in various cellular functions. They become integrated into cell membranes, where they exert influence over membrane fluidity and flexibility. This, in turn, has a direct impact on the membrane's ability to function effectively in processes, such as signal transduction, the transport of molecules in and out of the cell, and the regulation of enzymes bound to the membrane [84].

PUFAs are highly susceptible to lipid peroxidation due to the presence of weak C-H bonds at the bis-allylic positions. Recent research has primarily focused on ω-6 PUFAs (e.g., linoleic acid, gamma-linolenic acid, dihomo-gamma-linolenic acid, arachidonic acid, and adrenic acid) and ω-3 PUFAs (e.g., alpha-linolenic acid, eicosapentaenoic acid, and docosahexaenoic acid) in the context of ferroptosis. Among these, arachidonic acid and adrenic acid are the primary substrates of lipid peroxidation during ferroptosis. It's important to note that free PUFAs themselves do not directly drive ferroptosis; they must be esterified into membrane phosphatidylethanolamines or cholesteryl esters to become lethal after peroxidation [84].

**Fig. 2:**
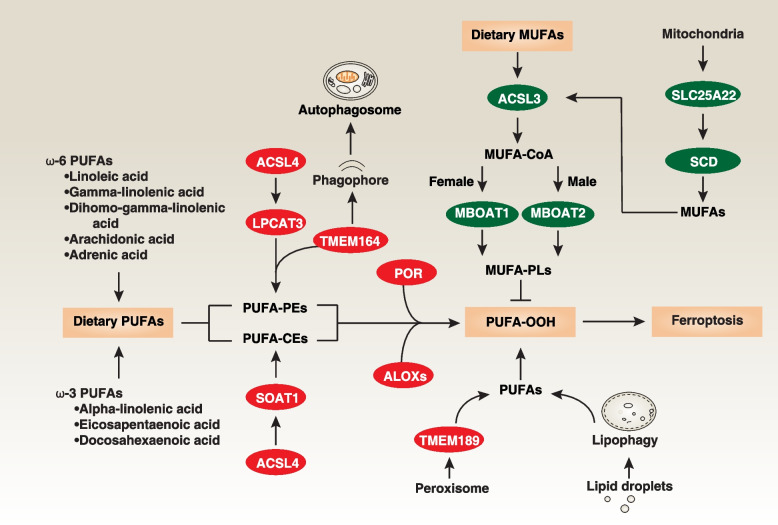
PUFAs and MUFAs in ferroptosis. Both dietary and de novo synthesized PUFAs play a central role in propagating lipid peroxidation during ferroptosis. Notably, free PUFAs themselves do not directly initiate ferroptosis; they must first undergo esterification into membrane PEs (phosphatidylethanolamines) or CEs (cholesteryl esters) following peroxidation to become lethal. ACSL4 regulates PUFA-PE synthesis through LPCAT3 and PUFA-CE synthesis via SOAT1. TMEM164 forms ferroptotic C20:4 ether phospholipids or induces autophagy, whereas TMEM189 is involved in peroxisome-mediated plasmalogen biosynthesis for lipid peroxidation. The POR and ALOX families mediate lipid peroxidation, leading to the production of PUFA-OOH, whereas ACSL3-mediated metabolism of MUFAs can exert an inhibitory effect on ferroptosis. The mitochondrial transporter SLC25A22 inhibits ferroptosis, in part, through the de novo synthesis of MUFAs. Furthermore, in ER^+^ breast cancer and AR^+^ prostate cancer cells, MBOAT1 and MBOAT2, upregulated by sex hormone receptors, inhibit ferroptosis through the production of MUFA-PLs, respectively

The downstream pathways mediated by ACSL4 (acyl-CoA synthetase long chain family member 4) result in the production of different PUFA-related acyl-CoA esters [85–87] (Fig. 2), even though ACSL4-independent ferroptosis can still occur [88, 89]. One pathway involves LPCAT3 (lysophosphatidylcholine acyltransferase 3), which incorporates PUFA into phosphatidylethanolamines in various types of cancer cells [85, 90]. The other pathway involves the activation of SOAT1 (sterol O-acyltransferase 1) in pancreatic cancer and leukemia cells, leading to the production of PUFA-cholesteryl esters instead of PUFA-phosphatidylethanolamines [91]. Both pathways contribute to lipid peroxidation, with PUFA-related acyl-CoA esters serving as substrates. The activity of ACSL4 can be enhanced by PRKCB/PKCβII (protein kinase C beta) in MDA-MB-231 (breast cancer cell line) and HT1080 cells, which catalyzes the phosphorylation of ACSL4 at Thr328 [92]. This suggests that ACSL4 phosphorylation is a crucial post-translational modification for regulating ferroptosis sensitivity.

Additional sources of PUFAs for lipid peroxidation include the peroxisome-mediated biosynthesis of plasmalogens through TMEM189 (transmembrane protein 189) in human ovarian and kidney cancer cell lines [93, 94] and lipophagy-mediated lipid droplet degradation in human liver cancer cell lines [95, 96], although the initiation signals for these alternative pathways are not well understood. Recently, TMEM164 (transmembrane protein 164) was identified as a mediator of ferroptosis, with multifunctional roles as an acyltransferase involved in the synthesis of C20:4 ether phospholipids in renal carcinoma 786-O cells [97] or in driving the formation of phagophores, leading to excessive autophagosome formation in HT1080 and pancreatic cancer cell line PANC1 [98]. These diverse sources of PUFAs during ferroptotic stimuli highlight the heterogeneity and plasticity of ferroptosis machinery.

Notably, not all lipids contribute to increased lipid peroxidation. In contrast, ACSL3 (acyl-CoA synthetase long chain family member 3)-mediated metabolism of MUFAs (monounsaturated fatty acids) can inhibit ferroptosis, possibly due to its structural differences from PUFAs [7, 99–101] (Fig. 2). The mitochondrial glutamate transporter SLC25A22 (solute carrier family 25 member 22) inhibits ferroptosis in pancreatic cancer cells by remodeling lipid metabolism pathways and promoting the production of GSH and MUFAs [102]. SLC25A22 achieves this by utilizing NADPH and stimulating the activity of the enzyme SCD/SCD1 (stearoyl-CoA desaturase) [102]. Moreover, MBOAT1 (membrane-bound O-acyltransferase domain-containing 1) and MBOAT2 (membrane-bound O-acyltransferase domain-containing 2), which are transcriptionally upregulated by sex hormone receptors, exert inhibitory effects on ferroptosis in ER^+^ breast cancer and AR^+^ prostate cancer cells, respectively [11]. They achieve this by remodeling the cellular phospholipid profile, ultimately leading to the synthesis of phospholipids rich in MUFA [11].

Hence, adjusting the balance between PUFA and MUFA levels is critical for regulating ferroptosis sensitivity. Nonetheless, lipid metabolism is a complex process with numerous interconnected steps. Understanding the bioavailability and absorption of PUFAs and MUFAs from diverse food sources and dietary supplements is vital, as it may play a context-dependent role in preventing or enhancing the ferroptotic process and its health implications.

### Lipid peroxidation

Lipid peroxidation stands as the defining characteristic of ferroptosis. The susceptibility of lipids to oxidation hinges on both the surrounding chemical environment and their inherent molecular structure. PUFAs are particularly prone to peroxidation due to the presence of bis-allylic moieties within their structure. The oxidation of PUFA during ferroptosis occurs through two primary mechanisms: enzymatic reactions and non-enzymatic autoxidation, driven by the Fenton reaction.

In enzymatic lipid peroxidation, the oxidation of PUFA is predominantly mediated by enzymes known as ALOXs and POR (Fig. 2). ALOXs, which are nonheme iron-containing enzymes, directly introduce molecular oxygen into PUFAs and PUFA-containing lipids within biological membranes. For instance, ALOX12 plays a crucial role in TP53-dependent ferroptosis [88]. ALOX15 is essential for ferroptosis induced by compounds (e.g., erastin or RSL3), as it forms a complex with PE binding protein 1 (PEBP1), specifically recognizing stearoyl-arachidonoyl-phosphatidylethanolamines and generating lipid peroxides [103]. Additionally, ALOXE3, ALOX5, ALOX12B, and ALOX15B have been implicated in ferroptosis induction, including in various cancer cells, in a context-dependent manner. Certain ALOX inhibitors (e.g., baicalein) protect cells against lipid peroxidation and ferroptosis [104]. However, it is important to note that genetic deletion of *Alox15* in *Gpx4* conditional knockout mice in kidney did not prevent ferroptosis *in vivo* [58]. Genetic deletion of *Alox12* and *Alox15* also failed to restore the viability of *Gpx4*-deficient T cells [59]. This suggests the existence of an ALOX-independent mechanism leading to ferroptosis in GPX4 knockout mice.

POR, on the other hand, directly provides electrons to P450 enzymes, facilitating the catalysis of PUFA peroxidation in an ALOX-independent manner in skin, ovarian, and kidney cancer cell lines [105, 106]. While POR expression is widespread, the expression of ALOX family members is highly cell- or tissue-specific. These observations collectively underscore the role of various iron-dependent enzymes, including ALOX and POR, in promoting lipid peroxidation and ferroptosis. Further investigations are necessary to explore the potential involvement of other oxygenases, such as cyclooxygenases and peroxygenases, in lipid peroxidation.

Nonenzymatic peroxidation of lipids is catalyzed by redox-active metals, with iron playing a prominent role. In non-enzymatic lipid peroxidation, when initial PLOOH (phospholipid hydroperoxides) are generated and not promptly reduced by the enzyme GPX4, they can react with ferrous ions, leading to the generation of alkoxyl and peroxyl radicals through the Fenton reaction. This process drives the propagation of PLOOH production to neighboring PUFA-phospholipids [23]. Furthermore, these toxic lipid products can react with nucleophilic amino acid residues within various proteins, forming covalent adducts and inducing protein lipoxidation [107]. If the defense system cannot effectively remove these toxic products, it can result in an irreversible point of no return in the progression of ferroptosis. Elucidating the precise mechanisms involved in this intricate process necessitates further investigation.

### Negative regulators

The defense against ferroptosis encompasses various antioxidant systems and membrane repair mechanisms, all working in concert to interrupt lipid peroxidation reactions or eliminate compromised membranes.

### GPX4-dependent antioxidant pathway

**Fig. 3:**
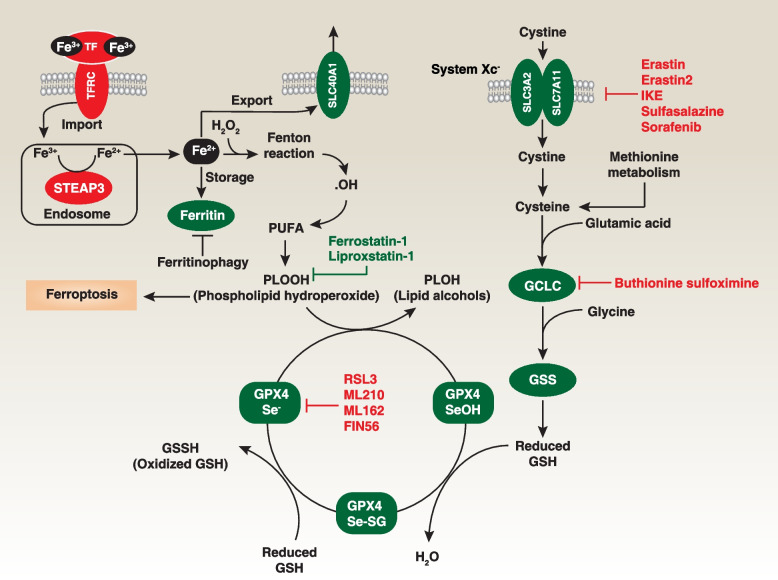
GPX4-dependent pathway in ferroptosis. The GPX4-dependent antiferroptotic pathway is associated with classical ferroptosis, a form of cell death driven by iron-dependent lipid peroxidation of PUFA. In this process, Fe^3+^ is transported into the intracellular space through binding to TF (transferrin) and its receptor TFRC. Within endosomes, STEAP3 converts intracellular Fe^3+^ to Fe^2+^, which can catalyze the production of .OH through the Fenton reaction, leading to lipid peroxidation. Conversely, storing Fe^2+^ in ferritin or exporting iron into the extracellular space via SLC40A1 can inhibit ferroptosis. Furthermore, the degradation of ferritin through ferritinophagy increases free Fe^2+^ levels, thereby promoting ferroptosis. GPX4 plays a pivotal role in this pathway by catalyzing the reduction of toxic PLOOH to non-toxic PLOH. The activity of GPX4 is tightly regulated by GSH (glutathione) and Se (selenium). Intracellular GSH levels are primarily controlled by system xc^-^-mediated cystine uptake and subsequent cysteine transformation for GSH synthesis. In addition to the system xc^-^, methionine metabolism serves as an additional intracellular source of cysteine for the synthesis of GSH. The radical-trapping antioxidants ferrostatin-1 and liproxstatin-1 are the most used ferroptosis inhibitors

GPX4 stands out as the exclusive member within the GPX family recognized for its role as a phospholipid hydroperoxidase, directly transforming PLOOH into the corresponding PLOH (phospholipid alcohols) [108]. This distinctive function establishes GPX4 as the pivotal negative regulator of ferroptosis [9]. The catalytic mechanism employed by GPX4 adheres to a ping-pong mechanism, characterized by the dynamic interchange of the enzyme's active site between oxidation and reduction states (Fig. 3).

Initially, GPX4's active site selenol (GPX4-SeH) undergoes oxidation by PLOOH, resulting in the formation of a selenenic acid intermediate (GPX4-SeOH). Subsequently, this intermediate interacts with GSH, leading to the creation of a selenium-glutathione adduct (GPX4-Se-SG) [10]. The system xc^-^, which plays a role in importing cysteine for GSH synthesis, is a significant upstream modulator in this process. Finally, through a reaction with a second GSH molecule, GPX4-Se-SG is converted back to GPX4-SeH, simultaneously generating oxidized glutathione (GSSG). A crystal structure analysis of seleno-GPX4 has unveiled the presence of seleninic acid (GPX4-Se-OO^-^) within the enzyme's active site [109]. This finding suggests an alternative reaction mechanism characterized by three distinct redox states (GPX4-SeH, GPX4-SeOH, and GPX4-Se-OO^-^) involving the catalytically active selenocysteine. These studies underscore the role of selenocysteine in GPX4 expression and activity [10].

To prevent hydroperoxide-induced ferroptosis, it is imperative to substitute selenocysteine with a cysteine residue (U46C) in GPX4 [110]. Furthermore, the R152H mutation in GPX4 can lead to Sedaghatian-type spinal metaphyseal dysplasia, a rare and fatal disease in newborns [111]. *In vitro* studies suggest that this R152H mutation doesn't directly impact the enzyme's catalytic activity but rather interferes with its allosteric activation by cardiolipin [112]. In the context of ferroptosis, the expression of GPX4 protein is regulated by either the ubiquitin-proteasome system or the autophagy degradation pathway [36, 113–117]. CKB (creatine kinase B)-mediated phosphorylation of GPX4 at serine residue 104 inhibits autophagy-mediated GPX4 degradation and subsequent ferroptosis in human liver cancer cells [116]. However, the exact checkpoint governing these two degradation pathways of GPX4 remains unclear.

GPX4 exists in three isoforms: mitochondrial, cytosolic, and nuclear GPX4 [108]. All of these isoforms are encoded by the same GPX4 gene, but have different transcription initiation sites. When the cytosolic GPX4 is genetically ablated or rendered inactive, it leads to early embryonic lethality [118]. Interestingly, reintroducing cytoplasmic GPX4, rather than its mitochondrial or nuclear counterparts, can rescue the lethal phenotype in *Gpx4*-null mice [119]. Generally, cytosolic GPX4 plays a prominent role in preventing ferroptosis. However, the question of whether mitochondrial GPX4 inhibits ferroptosis remains a subject of debate [13, 120, 121], and there is currently no research establishing the role of nuclear GPX4 in ferroptosis.

Global *Gpx4* knockout mice do not survive, but the phenotype of conditional *Gpx4* knockout mice depends on the specific cell or tissue involved. For instance, the conditional knockout of *Gpx4* in kidney cells, T cells, or B cells can induce ferroptotic damage, which can be reversed by supplementing with vitamin E or the ferroptosis inhibitor liproxstatin-1 [58, 59, 122, 123]. Conversely, the conditional knockout of *Gpx4* in myeloid cells, blood progenitor cells, and sperm cells can increase pyroptosis, necroptosis, and apoptosis in response to specific stresses [120, 124, 125]. Therefore, the antioxidant function mediated by GPX4 plays a context-dependent role in both ferroptosis and non-ferroptotic cell death.

### GPX4-independent antioxidant pathway

**Fig. 4:**
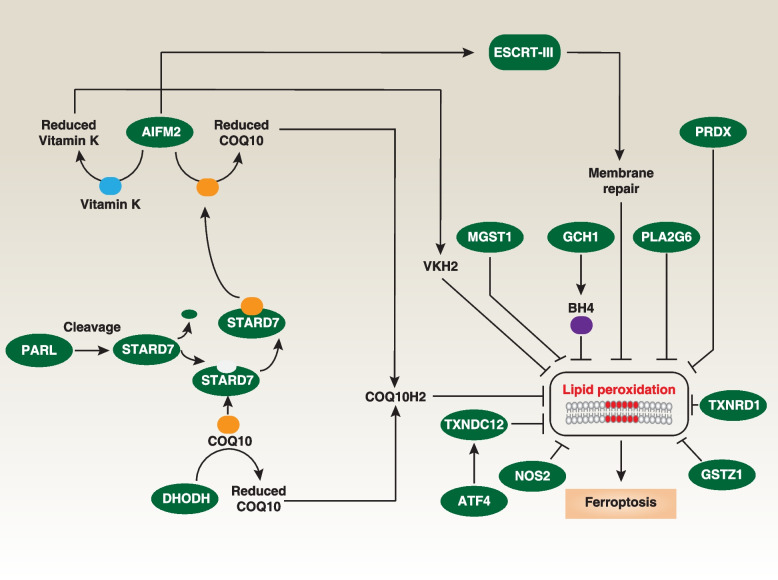
GPX4-independent pathway in ferroptosis. Organisms possess intricate networks of antioxidant and enzyme systems that collaborate to prevent lipid peroxidation-mediated ferroptosis. Apart from GPX4, several GPX4-independent pathways and proteins are involved. Specifically, AIFM2 (also known as FSP1) and DHODH act as ferroptosis suppressors by reducing COQ10 to COQ10H2. This process is facilitated by STARD7. The PARL-mediated cleavage of STARD7 occurs during its import into mitochondria, leading to the release of a portion of mature STARD7 into the cytosol. This cytosolic fraction delivers mitochondria COQ10 to the plasma membrane. AIFM2 also inhibits ferroptosis by either generating reduced vitamin K or activating the ESCRT-III membrane repair pathway. Furthermore, ER stress can induce ATF4-dependent expression of TXNDC12, which serves to limit lipid peroxidation

Although the ER (endoplasmic reticulum) has been suggested as the primary site for the initiation of ferroptosis [126], various cellular compartments contribute to the ferroptosis process [127]. Consequently, different antioxidant systems possess unique capabilities and operate within distinct cellular locales to inhibit ferroptosis. GPX4 has a prominent function within the cytosol, actively inhibiting ferroptosis. Simultaneously, various GPX4-independent enzymes contribute to ferroptosis resistance in distinct cellular compartments (Fig. 4), primarily by generating radical-trapping antioxidants, with a notable emphasis on COQ10 (coenzyme Q10).

AIFM2/FSP1 (apoptosis inducing factor mitochondria associated 2), a member of the apoptosis-inducing factor family located in mitochondria, has garnered attention as a potent suppressor of ferroptosis in diverse cancer cells, including HT1080 [12, 15]. It achieves this by translocating from mitochondria to the cell membrane and participating in the reduction of COQ10 to COQ10H2. STARD7 (StAR-related lipid transfer domain-containing 7), located in the intermembrane space of mitochondria and in the cytosol after cleavage by the rhomboid protease PARL (presenilin-associated rhomboid-like), is involved in transporting COQ10 to the plasma membrane AIFM2, thus suppressing ferroptosis in HeLa (human cervical cancer cell line) and HCT116 (human colorectal cancer cell line) cells [128]. Furthermore, AIFM2 contributes to the inhibition of ferroptosis by engaging in membrane repair in pancreatic cancer cells (PANC1) and liver cancer cells (HepG2) [129] and is also involved in the reduction of vitamin K in 786-O (human renal carcinoma cell line) and A375 (human melanoma cell line) cells, which includes warfarin-resistant vitamin K reduction [14, 130, 131]. The involvement of AIFM2 in ferroptosis is influenced by phase separation, a process in which a homogeneous mixture of substances spontaneously separates into distinct phases, and can be initiated through N-terminal myristoylation, which is facilitated by a compound called icFSP1 in a variety of human cancer cells [14].

DHODH (dihydroorotate dehydrogenase (quinone)), a flavin-dependent mitochondrial enzyme involved in de novo pyrimidine biosynthesis, has garnered attention as a potential therapeutic target for various diseases. DHODH inhibitors (e.g., BAY 2402234, PF-06726209, and brequinar) have been developed for potential use in cancer treatment and immunosuppression. The activity of DHODH influences the vulnerability of cancer cells with low GPX4 levels to ferroptosis, likely due to DHODH-mediated utilization of COQ10 as an electron acceptor in HT1080 and NCI-H226 (human lung cancer cell line) cells [13]. Inhibiting DHODH activity leads to a reduction in COQ10 levels, compromising antioxidant capacity and increasing susceptibility to lipid peroxidation and ferroptosis [13]. Nonetheless, there is an ongoing debate regarding the potential unintended effects of the DHODH inhibitor brequinar on AIFM2 in cancer cells [132, 133].

TXNDC12 (thioredoxin domain-containing protein 12), also known as ERp18 or ERp19, plays a role in inhibiting ferroptosis independently of GPX4 in human leukemia cells within the ER [134]. Its expression is increased in human leukemia cells during ferroptosis due to the activation of transcription factor ATF4 (activating transcription factor 4) [134]. Solid cancer cells with higher baseline TXNDC12 expression tend to exhibit resistance to ferroptosis [135]. Conversely, genetic knockdown of *TXNDC12* enhances ferroptosis sensitivity, leading to increased lipid peroxidation both i*n vitro* and *in vivo* [134, 135]. Hence, targeting TXNDC12 could prove to be a valuable strategy in cancer therapy.

Redundancy in antioxidant systems acts as a backup mechanism to safeguard cellular integrity and function in the face of oxidative stress, reducing the risk of damage. Other antioxidant enzymes, including GCH1 (GTP cyclohydrolase 1) [136], NOS2/iNOS (nitric oxide synthase 2) [137], MGST1 (microsomal glutathione S-transferase 1) [138], GSTZ1/maleylacetoacetate isomerase (glutathione S-transferase zeta 1), TXNRD1 (thioredoxin reductase 1) [139], PLA2G6/iPLA2β/PNPLA9 (phospholipase A2 group VI) [16, 17], and peroxiredoxins (PRDX) [140], also inhibit ferroptosis in a context-dependent manner, even in *GPX4*- or *AIFM2*-knockout cancer cells. Each of these pathways plays a unique and complementary role, collectively providing comprehensive protection against oxidative damage during ferroptosis.

Metal ions, particularly iron, play pivotal roles in initiating oxidative stress and triggering ferroptosis. To counteract these detrimental reactions, cells employ a range of metal-binding proteins, such as transferrin (TF) and ferritin, which efficiently sequester free iron [77, 78]. Intracellular metal equilibrium is meticulously upheld by specialized proteins, including metal chaperones that are tasked with delivering metals to their intended protein targets. Metallothioneins, a family of cysteine-rich proteins, also contribute significantly to regulating the availability of metal ions, thereby reducing their participation in oxidative damage and the ferroptosis process in human liver cancer cells (HepG2) [141]. Furthermore, certain clinical drugs with metal-chelating properties have shown promise in preventing ferroptosis by interfering with iron-related processes. Examples of these drugs include deferoxamine, deferiprone, deferasirox, and ciclopirox [142]. These compounds hold potential as therapeutic agents that can mitigate the impact of iron-mediated oxidative stress, thereby offering a new avenue for intervention in diseases where ferroptosis plays a significant role.

### Membrane repair system

The membrane repair system operates to mitigate and reverse damage to the plasma membrane resulting from mechanical stress, injury, or disruption. When the membrane is compromised, cellular mechanisms are activated to restore its integrity, thereby reducing the risk of ferroptosis.

Ca^2+^ plays a central role in initiating membrane repair. Following plasma membrane damage, Ca^2+^ enters the cytoplasm from extracellular sources, serving as a signal to trigger downstream repair processes such as ESCRT (endosomal sorting complexes required for transport)-III and exocytosis. Ca^2+^ signaling from different organelles has a dual impact on ferroptosis sensitivity, highlighting the need for timely signal monitoring.

During cellular stress, which includes oxidative stress associated with ferroptosis, ESCRT-III components are mobilized to sites of membrane damage, where they play a critical role in facilitating repair and preventing irreversible damage that can lead to ferroptosis [143]. For instance, when there is an influx of Ca^2+^ in pancreatic cancer cells, specific subunits of ESCRT-III, referred to as CHMPs (charged multivesicular body proteins), particularly CHMP5 and CHMP6, are recruited and assembled at the damaged site, actively contributing to the restoration of membrane integrity in liver and pancreatic cancer cells [143]. In contrast, the knockout of either *CHMP5* or *CHMP6* increases the susceptibility of pancreatic cancer cells (PANC1) to ferroptosis [143].

Exocytosis constitutes another crucial aspect of the membrane repair system, where intracellular vesicles fuse with the plasma membrane to facilitate repair. This essential process, involved in the transport and secretion of diverse molecules, including proteins, lipids, neurotransmitters, and hormones, plays a protective role. It particularly safeguards neurons from ferroptosis by facilitating the release of lipids and iron from autolysosomes [144].

While repair mechanisms in the plasma membrane are well-studied, it remains unclear whether organelles employ similar mechanisms for repair. Further exploration of these mechanisms and their associated signaling pathways has the potential to pave the way for the development of targeted therapies aimed at addressing diseases associated with impaired ferroptosis. Additionally, the emerging field of understanding the role of membrane repair in immune responses, including inflammation and tissue repair, holds great promise and continues to garner interest.

## Advantages of ferroptosis-based antitumor therapy

Extensive studies suggest that ferroptosis plays a pivotal role in tumor suppression, thus providing new opportunities for cancer therapy.

### Personalized medicine

Identifying specific vulnerabilities in cancer cells that make them susceptible to ferroptosis allows for more personalized treatment, potentially improving outcomes. Below, we highlight three key gene mutation signals in the regulation of ferroptosis sensitivity.

*RAS* mutations, commonly found in various cancer types, promote abnormal cell growth and resistance to certain therapies. Ferroptosis, initially recognized as a targeted therapy for *RAS*-mutated cancer cells, offers selectivity in eliminating them while sparing normal cells [4]. Blocking the RAS pathway or its downstream RAF-MEK-ERK axis can reverse the specific cytotoxic effects induced by erastin [145]. Additionally, mutations in *EGFR* (epidermal growth factor receptor), upstream of RAS, enhance the susceptibility to ferroptosis induced by cystine depletion [146]. This susceptibility is likely attributed to the activation of the RAS-MAPK axis mediated by mutant *EGFR* in non-small cell lung cancer cells [146]. A recent study demonstrated that mutant *KRAS* elevates AIFM2 levels by activating MAPK and NFE2L2/NRF2 (NFE2 like BZIP transcription factor 2), thereby suppressing ferroptosis in pancreatic cancer cells [147]. Combining the induction of ferroptosis with the inhibition of defense mechanisms, such as AIFM2 or NFE2L2 inhibitors, holds promise as a potential treatment strategy for pancreatic cancers with *KRAS* mutations. In a similar fashion, mutant *KRAS* can activate NFE2L2-dependent expression of SLC7A11, resulting in elevated intracellular cysteine levels and increased GSH biosynthesis [148]. Inhibition of SLC7A11 by erastin can induce synthetic lethality in *KRAS*-mutated lung adenocarcinoma cells [148]. Taken together, these findings indicate that cancer cells harboring *KRAS* mutations are susceptible to ferroptosis induction.

Subsequent studies have revealed vulnerabilities associated with gene mutations, particularly *TP53*, impacting ferroptosis sensitivity. TP53, a tumor suppressor protein known as the 'guardian of the genome,' plays a crucial role in maintaining genomic stability and regulating cell cycle progression. For example, the acetylation-deficient *TP53* variant, TP53[3KR], lacks the ability to induce apoptosis and cell cycle arrest but retains its tumor suppression function akin to wild-type TP53. It achieves this by suppressing SLC7A11 expression, leading to ferroptosis induction in various cancer cells [149, 150]. TP53 also influences ferroptosis sensitivity through other mechanisms, such as downregulating VKORC1L1 (vitamin K epoxide reductase complex subunit 1 like 1) in vitamin K metabolism [151] and inducing SAT1 (spermidine/spermine N1-acetyltransferase 1), an enzyme in polyamine catabolism that produces ROS [152]. Conversely, TP53 can inhibit ferroptosis in certain conditions. For example, *TP53* deletion in colorectal cancer cells increases ferroptosis sensitivity by activating DPP4-mediated NOX1 pathway [70]. *TP53* mutations, such as R175H, can lead to modified TP53 proteins that act as suppressors of ferroptosis by preventing BACH1 (BTB domain and CNC homolog 1)-mediated downregulation of SLC7A11, thus promoting tumor growth [153]. These findings underscore the varied roles of TP53 in regulating ferroptosis sensitivity, which can vary depending on the specific tumor types.

KEAP1 (kelch like ECH associated protein 1), an enzyme often mutated in lung cancer, interacts with NFE2L2, leading to NFE2L2 degradation [154, 155]. Mutations in *KEAP1* or the augmentation of SQSTM1 (sequestosome 1)-mediated KEAP1 protein degradation can enhance the stability of NFE2L2 protein, enabling cancer cells to evade ferroptosis and acquire drug resistance [34]. NFE2L2 plays a pivotal role in regulating antioxidant genes involved in both GPX4-dependent (e.g., GPX4 and SLC7A11) and GPX4 independent pathways (e.g., AIFM2 and MT1G [metallothionein 1G]) [34, 83, 156]. In TNBC (triple-negative breast cancer), methylation of KEAP1 by PRMT5 (protein arginine methyltransferase 5) results in decreased levels of NFE2L2 and its downstream target gene HMOX1 (heme oxygenase 1), thereby influencing the sensitivity of the cancer cells to ferroptosis [157]. These findings also demonstrate an epigenetic strategy for controlling ferroptosis sensitivity in TNBC cells by modulating the KEAP1-NFE2L2 pathway. p14ARF, also known as ARF tumor suppressor, is a protein product encoded by the alternate reading frame of the CDKN2A (cyclin dependent kinase inhibitor 2A) locus. p14ARF enhances ferroptosis, both with and without TP53, by inhibiting NFE2L2, particularly in *TP53*-knockout cells. Loss of p14ARF activates NFE2L2, aiding cancer cell survival during oxidative stress and ferroptosis [158]. Apart from KEAP1, other oncogenes, such as KRAS^G12D^ [147], BRAF^V619E^ [159], and MYC^ERT2^ [159], can increase NFE2L2 expression or stability, maintaining cellular antioxidant defenses and potentially defending against ferroptosis. Overall, dysregulation of NFE2L2 or its downstream pathways can lead to increased susceptibility to ferroptosis, making it an attractive target for research and potential therapeutic interventions in certain types of cancer.

In addition to genetic mutations, recent reviews have highlighted the influence of multiple tumor-related signaling pathways on ferroptosis sensitivity. These pathways, including MTOR (mechanistic target of rapamycin kinase) signaling [31, 43, 160], hypoxia response [161–163], the Hippo pathway [164, 165], and autophagic degradation [116, 166–172], play context-dependent roles in determining ferroptosis susceptibility. Moreover, bioinformatic analyses have uncovered aberrant expression patterns of ferroptosis regulator genes in cancer patients. The development of a ferroptosis potential index for patients offers a valuable tool for predicting disease progression, evaluating immune responses, and characterizing metabolic alterations.

### Overcoming drug resistance

The persistence of cancer cells, including drug-tolerant populations, presents a substantial challenge in both preclinical research and clinical practice. Some cancer cells resist conventional therapies due to apoptosis deficiencies, rendering them resistant to elimination. Ferroptosis-based therapies show promise in targeting vulnerabilities unique to these resistant cells, offering a potential solution.

Acquired drug resistance frequently hinders cancer treatments from achieving stable and complete responses. Recent evidence highlights the significance of non-mutational drug resistance mechanisms in the survival of residual cancer 'persister' cells [173]. These persister cells act as a reservoir from which drug-resistant tumors can potentially emerge, making targeting them an attractive therapeutic opportunity to prevent tumor relapse. Cancer cells in a high mesenchymal therapy-resistant state rely on the lipid hydroperoxidase GPX4 for survival [174, 175]. Preclinical studies have demonstrated the efficacy of GPX4 inhibitors, such as RSL3 or ML210, in eliminating persister cancer cells *in vitro* and in xenograft models [174, 175].

In addition, there is a scarcity of ferroptosis activators capable of targeting cancer stem cell niches and populations of cells that inherently tolerate therapy or acquire drug resistance [176–178]. These findings support the potential clinical utility of GPX4 inhibitors to prevent tumor relapse or to combat cancer cells that can adopt a therapy-resistant state.

### Complementary to other therapies

Ferroptosis-based treatments can be employed in combination with existing cancer therapies (e.g., chemotherapy, radiotherapy, or immunotherapy) to potentially enhance overall treatment efficacy. Combining ferroptosis activators with traditional chemotherapeutic drugs (e.g., temozolomide, oxaliplatin, cisplatin, gemcitabine, and 5-fluorouracil) or targeted drugs (e.g., olaparib, cetuximab, and sunitinib) may help overcome drug resistance or enhance their effects. Radiotherapy itself can induce ferroptosis in tumor cells, and the addition of ferroptosis agonists can improve radiation therapy outcomes in tumor models. For instance, *in vitro* and *in vivo* studies using cancer cell lines (HT1080, HeLa, NCI-H1975 [human non-small cell lung cancer cell line], and B16F10 [human melanoma cell line]) demonstrated that combining ferroptosis activators with radiation therapy reduced tumor growth compared to radiation therapy alone [179–182]. Mechanistically, this combination therapy not only inhibits the expression of anti-ferroptotic proteins, such as GPX4 and SLC7A11, but also induces the expression of ACSL4, leading to increased lipid peroxidation and ferroptosis [183]. In cancer cells, PARP1 (poly(ADP-Ribose) polymerase 1) and PARP2 can activate DNA repair pathways to prevent DNA damage and cell death caused by radiotherapy. When radiotherapy is combined with PARP inhibitors (e.g., niraparib), it can induce DNA damage and activate the CGAS (cyclic GMP-AMP synthase)-STING1 (stimulator of interferon response cGAMP interactor 1) pathway. This activation, in turn, triggers tumor ferroptosis and subsequently stimulates CD8^+^ T cells-mediated antitumor immunity in colorectal cancer models [184]. In addition to its benefits in inhibiting tumor growth, ACSL4-dependent ferroptosis is implicated in radiation-induced intestinal injury, a common gastrointestinal complication resulting from radiotherapy [185]. This highlights that ferroptosis can also be a side effect of radiation-induced damage in normal tissues.

**Fig. 5:**
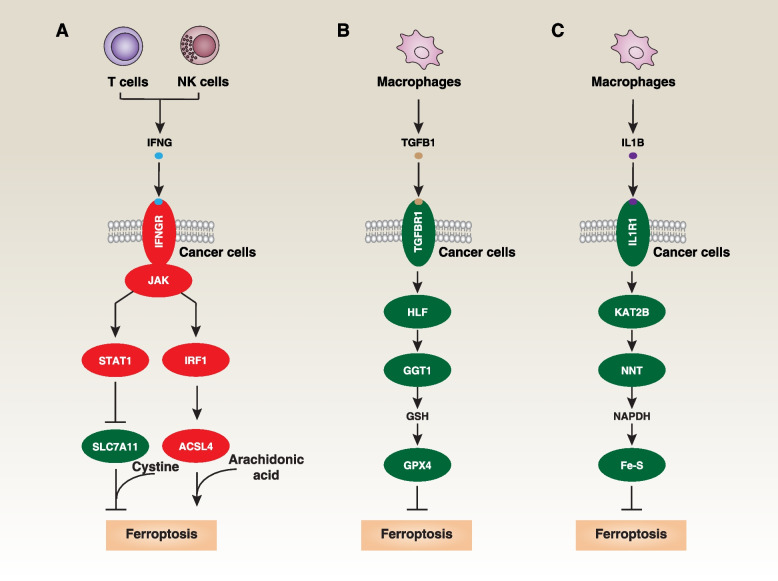
Effects of cytokines produced by immune cells on ferroptosis in cancer cells. **A** IFNG production by CD8^+^ T cells or natural killer cells can promote ferroptosis in cancer cells through STAT1-dependent downregulation of SLC7A11 or IRF1-dependent upregulation of ACSL4. **B** TGFB1 production by macrophages can inhibit ferroptosis in cancer cells by enhancing HLF-dependent GSH synthesis through GGT1, ultimately leading to the activation of GPX4. **C** IL1B production by macrophages can inhibit ferroptotis in cancer cells through KAT2B-dependent acetylation. This acetylation enhances NNT's affinity for NADP^+^ and leads to increased production of NADPH, thus promoting the maintenance of Fe‐S clusters

The sensitivity of ferroptotic cancer cell death is influenced by the release of cytokines from immune cells. IFNG/IFN-γ (interferon gamma), a versatile cytokine released by CD8^+^ T cells or NK (natural killer) cells, is a crucial effector molecule in immunotherapy. IFNG can enhance the cell death of tumor cells (HT1080, A375, and ID8 [murine ovarian surface epithelial cell line]) induced by ferroptosis activators, such as erastin, RSL3, or cysteine depletion [186]. This effect is partly achieved by downregulating SLC7A11 through its receptor and activating the JAK (Janus kinase)-STAT1 (signal transducer and activator of transcription 1) pathway in cancer cells [186] (Fig. 5A). In addition, IFNG stimulates ACSL4 expression through an IRF1 (interferon regulatory factor 1)-dependent mechanism, thereby augmenting arachidonic acid-related ferroptosis in animal models and impeding tumor growth [187] (Fig. 5A). Combining ferroptosis inducers with immune checkpoint inhibitors, such as anti-CTLA4 (cytotoxic T-lymphocyte associated protein 4), anti-PDCD1/PD-1 (programmed cell death 1), or anti-CD274/PD-L1 (programmed death ligand 1) antibodies, enhances tumor suppression compared to using immune checkpoint inhibitors alone. Conversely, tumor-associated macrophage-derived TGFB1/TGFβ1 (transforming growth factor beta 1) suppresses ferroptosis through the activation of the HLF (HLF transcription factor, PAR BZIP family member)-GGT1 (gamma-glutamyltransferase 1)-GSH-GPX4 pathway in TNBC cells [188] (Fig. 5B). Moreover, IL1B/IL-1β (interleukin 1 beta) stimulation leads to KAT2B (lysine acetyltransferase 2B)-dependent acetylation and activation of NNT (nicotinamide nucleotide transhydrogenase) in gastric cancer cells, resulting in increased production of NADPH and the preservation of Fe-S clusters within mitochondria [189] (Fig. 5C). This mechanism functions to shield tumor cells from ferroptosis and immune-based therapies. However, IL1B is also a mediator of immunogenic cell death driven by pyroptotic cell death [190], adding complexity to the feedback mechanisms involving different cell death and immune mediators.

### Side effects of ferroptosis-based antitumor therapy

There is growing evidence that current ferroptosis activators can trigger cell death in normal cells, resulting in adverse effects during cancer therapy.

### Immune cell death

**Fig. 6:**
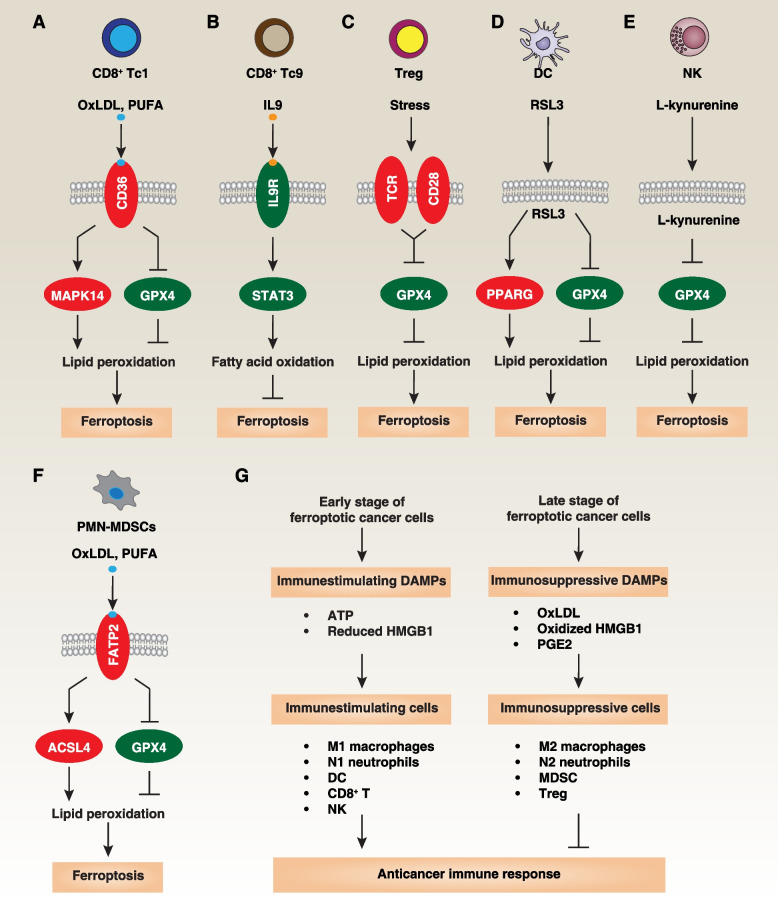
Mechanisms of ferroptosis in immune cells and the effects of DAMP on ferroptosis-related tumor immunity. **A-F** Mechanisms of ferroptosis in immune cells within the tumor microenvironment. **G** The impact of ferroptotic cancer cells on tumor immunity, which depends on the stage of cell death, DAMP release, and the infiltration of immune cells within the tumor microenvironment

Recent studies have shown that classical ferroptosis activators or oxidized lipids can inadvertently lead to the death of these crucial antitumor immune cells. Among these immune cells are cytotoxic CD8^+^ T cells, specialized in directly recognizing and eliminating cancer cells by targeting cancer-specific antigens on their surfaces. Inducing ferroptosis in CD8^+^ T cells has been found to dampen antitumor immunity in mouse models of clone cancer (using B16 or MC38 cell line) in B6 mice [191, 192] (Fig. 6A). This effect is linked to the expression of CD36, a cell surface protein known for its roles in lipid metabolism, including fatty acid and lipoprotein uptake, as well as its involvement in innate immunity and inflammation. The uptake of OxLDL (oxidized low-density lipoproteins) or PUFA by CD36 can trigger GPX4 downregulation and MAPK14/p38 (mitogen-activated protein kinase 14) phosphorylation, ultimately leading to lipid peroxidation and ferroptosis or functional exhaustion of cytotoxic CD8^+^ T cells in mouse models of clone cancer in B6 mice [191, 192]. These effects can be prevented by ferroptosis inhibitors or by *CD36* deficiency [191, 192]. GPX4 is not essential for T cell development, but plays a crucial role in maintaining T cell homeostasis and supporting T cell-dependent immune responses during acute viral infections [59]. Moreover, overexpressing *GPX4 *or depleting *CD36 *can rescue effector functions of cytotoxic CD8^+^ T cells, enhancing their ability to control tumor growth deficiency [191, 192]. CD8^+^ T cells can be categorized into various functional subsets, including Tc1 cells that produce high amounts of IFNG and Tc9 cells that produce IL9 (interleukin 9) at lower levels of IFNG. Unlike conventional cytotoxic CD8^+^ Tc1 cells, CD8^+^ Tc9 cells activate the IL9-STAT3-fatty acid oxidation pathway in B16 tumor–bearing Thy1.2^+^ B6 mice [193] (Fig. 6B). This pathway provides protection against ROS-induced lipid peroxidation and ferroptosis in the tumor microenvironment [193]. Furthermore, *Gpx4*-deficient Treg (T regulatory) cells are vulnerable to ferroptosis and exhibit elevated IL1B production, which, in turn, enhances T Th17 (helper cell 17) responses in B6 mice inoculated with B16.F10 melanoma cells [194] (Fig. 6C). This impairment compromises Treg-mediated immunosuppression within the tumor microenvironment, ultimately restricting *in vivo* tumor growth [194].

DCs (dendritic cells) are another type of immune cell that contributes to antitumor immunity by activating cytotoxic T cells. Interestingly, the GPX4 inhibitor RSL3 can impair the maturation and function of DCs in tumor suppression by activating PPARG/PPARγ (peroxisome oroliferator activated receptor gamma)-dependent ferroptosis in DC2.4 cell line [195] (Fig. 6D). Early-stage ferroptosis in the MCA205 mouse fibrosarcoma cell line treated by RSL3 for 1 hour exhibits immunogenicity when exposed to activated DCs, in contrast to late-stage ferroptosis induced by ML162 for 14 hours in the MCA205 mouse fibrosarcoma cell line [196, 197]. The potential mechanism responsible for these differences may involve a switch in the release of immunostimulatory danger/damage-associated molecular patterns (DAMPs) (e.g., ATP and reduced HMGB1 [high mobility group box 1]) to immune-suppressive DAMPs (e.g., oxidized HMGB1 or PGE2 [prostaglandin E2]) [198, 199]. PGE2 is an immunosuppressive lipid that hinders the anti-tumor activity of cDC1 (conventional type 1 DC), NK cells, and effector T cells, while also activating immunosuppressive cells, such as MDSCs (myeloid-derived suppressor cells) and Tregs, promoting immune escape.

NK cells represent yet another class of immune cells capable of targeting and eliminating cancer cells without prior sensitization or antigen recognition. However, tumor-associated NK cells within the tumor microenvironment of ovarian cancer patients exhibit increased expression of proteins related to lipid peroxidation, oxidative damage, ferroptosis, as well as senescence and autophagy pathways [200]. These alterations impair the cytotoxic activity of NK cells against ovarian cancer cell OVCAR8 *in vitro* [200]. L-Kynurenine is a naturally occurring molecule and a critical intermediate in the tryptophan metabolic pathway. In the context of cancer and immunology, L-kynurenine has garnered attention for its potential immunosuppressive effects. A recent study revealed that L-kynurenine can contribute to immune tolerance by inducing lipid peroxidation and ferroptosis in NK cells, ultimately promoting gastric cancer growth in *in vivo* experiments [201] (Fig. 6E). Thus, the modulation of the tryptophan metabolic pathway contributes to the development of potential immunotherapeutic strategies by influencing NK cell death through ferroptosis.

Furthermore, pathologically activated neutrophils, known as PMN-MDSCs (myeloid-derived suppressor cells), act as major negative regulators of antitumor immunity. While PMN-MDSCs in the tumor microenvironment undergo spontaneous ferroptosis, this process leads to the release of oxygenated lipids (e.g., oxidized AA-PEox) or PGE2, limiting the activity of both human and mouse T cells [202] (Fig. 6F). Inhibition of ferroptosis using liproxstatin-1 results in PMNs' depletion and enhances the effectiveness of anti-PDCD1/PD-1 antibody treatment on pancreatic cancer cell growth in immunocompetent mice [202]. These observations highlight the significant impact of both the particular cell undergoing ferroptosis and the release of DAMP within the tumor microenvironment on the functionality of cytotoxic CD8^+^ T cells (Fig. 6G).

**Fig. 7:**
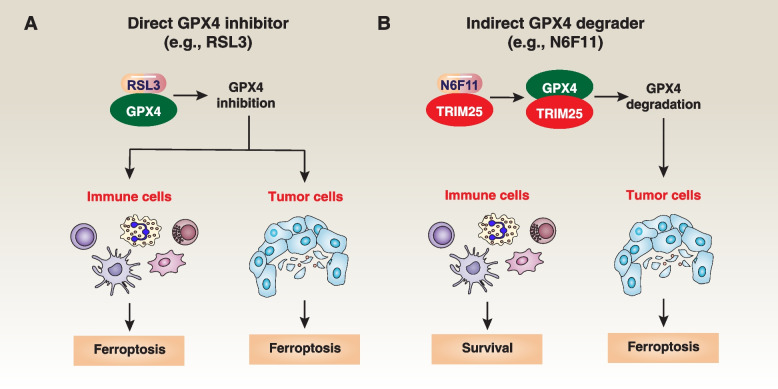
Strategy for GPX4 Inhibition. **A** Traditional GPX4 inhibitors, such as RSL3, lead to the loss of GPX4 activity. As GPX4 is widely expressed in both immune and cancer cells, they induce non-selective ferroptosis. **B** N6F11 can bind to TRIM25, subsequently triggering TRIM25-dependent GPX4 degradation to induce ferroptosis. Given that TRIM25 is mainly expressed in cancer cells, the use of N6F11 does not induce ferroptosis in non-cancer cells

To address this unintended consequence, it is crucial to develop strategies that can specifically target tumor cells while preserving the functionality of immune cells and their ability to surveil and combat cancer. In a recent study, a screening of a library containing more than 4000 small-molecule compounds identified N6F11 as the first cell-specific ferroptosis activator [36]. N6F11 selectively induces ferroptosis in cancer cells by targeting TRIM25 (tripartite motif containing 25)-mediated GPX4 degradation in pancreatic cells, both *in vitro* and in various mouse models, such as xenografts, orthotopic models, and transgenic models [36] (Fig. 7). TRIM25 is primarily expressed in cancer cells, rather than in immune cells (DC, T, NK, and neutrophil cells) [36]. This finding may represent a safe and effective approach to enhance ferroptosis-driven antitumor immunity. Additional research is needed to determine whether there are other TRIM25-dependent protein substrates involved in the selective induction of ferroptosis in cancer cells.

### Bone marrow impairment

Anticancer agents can disrupt the bone marrow's capacity to generate blood cells, potentially causing conditions, such as anemia, thrombocytopenia (low platelet count), or leukopenia (low white blood cell count). Inducing ferroptosis can lead to the death of stem cells and damage to the bone marrow, which may impact hematopoiesis and result in bone marrow suppression.

Recent research has shed light on the mechanisms involved. HSCs (hematopoietic stem cells) possess unique physiological adaptations that sustain lifelong blood cell production, including finely regulated protein synthesis rates. Disrupting MYSM1 (Myb like, SWIRM and MPN domains 1), a histone deubiquitinase, can compromise human HSC function, mimicking the bone marrow failure seen in patients, by triggering lipid peroxidation and ferroptosis [203]. Additionally, healthy HSCs are susceptible to ferroptosis induced by compounds such as erastin, FIN56, FINO2, and RSL3, primarily due to their low protein synthesis levels [203]. Therefore, inhibiting ferroptosis represents a potential strategy to augment HSC numbers, supporting the production of all blood cell types, including red blood cells (erythrocytes), white blood cells (leukocytes), and platelets (thrombocytes).

Furthermore, the activation of FANCD2 (fanconi anemia complementation group D2), a nuclear protein involved in DNA damage repair, plays a protective role against bone marrow injury caused by ferroptosis [204]. These discoveries suggest the existence of a nuclear protection pathway designed to alleviate ferroptotic damage in bone marrow dysfunction. Activators of FANCD2 have the potential to enhance its capacity to repair damaged DNA and shield cells from the detrimental consequences of DNA damage.

In light of these findings, monitoring the bone marrow and blood counts during ferroptosis-mediated therapy is crucial for assessing the impact of treatment on the hematological system.

### Liver and kidney damage

Many cancer treatments, including chemotherapy and targeted therapies, go through hepatic metabolism and renal elimination processes, which can increase the exposure of these organs to potentially harmful substances. As a result, the liver and kidneys may be at risk of damage. Similar to some other anticancer agents, specific ferroptosis activators have the potential to induce hepatotoxicity (liver damage) or nephrotoxicity (kidney damage).

Research has shown that when *Gpx4* is conditionally knocked out in the kidney or liver, it can lead to spontaneous ferroptotic damage [58, 205]. Fortunately, this damage can be reversed through treatment with vitamin E or liproxstatin-1. Therefore, there is a possibility that inhibition of GPX4, which promote ferroptosis, may have toxicity effects on the liver and kidneys [206].

To proactively manage this risk and ensure patient safety, it is crucial to closely monitor the function of the liver and kidneys throughout ferroptosis treatment. This can be achieved by conducting regular blood tests, including liver function tests and kidney function tests, such as measuring serum creatinine and blood urea nitrogen levels. These monitoring measures are indispensable for the early detection of any signs of damage or dysfunction in these vital organs.

### Cachexia

Cachexia is a complex metabolic syndrome often associated with chronic illnesses, such as cancer. It is characterized by symptoms, such as weight loss and muscle wasting [207]. Ferroptosis-based treatments can contribute to the development of cachexia.

In a study involving mice fed a high-fat, low-carbohydrate ketogenic diet, the induction of ferroptosis effectively suppressed tumor growth [208]. However, it also resulted in systemic effects, including increased lipid peroxidation and decreased levels of NADPH, leading to early-onset cachexia and reduced survival rates [208]. To address these systemic side effects, researchers employed a pulsed administration of glucocorticoids. This approach demonstrated the potential for improving therapeutic outcomes by gaining a deeper understanding of the mechanisms behind these side effects.

Furthermore, GDF15 (growth differentiation factor 15), a member of the TGFB superfamily produced by stressed cells, plays a role in reducing food intake by binding to its receptor GFRAL (GDNF family receptor alpha like) in the area postrema [208]. While GDF15 levels are elevated in cachexia resulting from ferroptosis, specific laboratory markers to distinguish it from other forms of cachexia have yet to be identified.

### Secondary tumorigenesis

Secondary tumorigenesis refers to the occurrence of new, unrelated tumors in individuals who have previously undergone cancer treatment. While cancer therapies are designed to target and eliminate cancer cells, they can inadvertently affect healthy cells and tissues, potentially leading to the development of new cancers. Although direct evidence is currently limited, several transgenic animal studies have suggested a potential role for ferroptotic damage in initiating tumorigenesis.

For instance, experiments involving high-iron diets or the depletion of *Gpx4*, which induces ferroptosis, promote pancreatitis and pancreatic tumorigenesis in mice [209, 210]. Conversely, inhibiting ferroptosis through the administration of liproxstatin-1 reduce spontaneous pancreatic cancer formation in a mouse model driven by *Kras*^*G12D*^ mutations, suggesting that ferroptosis may play a role in pancreatic cancer development [209, 210]. This ferroptosis-induced pancreatic cancer development could be linked to macrophage infiltration and polarization, mediated by the release of 8-OHG (8-hydroxy-2′-deoxyguanosine) resulting from ferroptotic damage and subsequent activation of the STING1-dependent DNA sensor pathway [209, 210]. Moreover, the oncogenic KRAS protein can be released by ferroptotic pancreatic cells, promoting pro-tumor M2 macrophage polarization through uptake by the AGER/RAGE (advanced glycosylation end-product specific receptor) [211]. In addition, the process of mitophagy-mediated degradation of mitochondrial iron importers (SLC25A37 and SLC25A28) results in elevated mitochondrial iron accumulation [212]. This, in turn, triggers the HIF1A (hypoxia inducible factor 1 subunit alpha)-dependent Warburg effect and AIM2 (absent in melanoma 2)-dependent inflammasome activation, contributing to *Kras*^*G12D*^-induced pancreatic tumorigenesis [212]. Thus, mitochondrial iron dysfunction can contribute to tumorigenesis through hypoxia and inflammasome signals. Some studies suggest that M1 macrophages exhibit greater resistance to ferroptosis compared to the M2 phenotype, despite similar expression levels of GPX4, ACSL4, and LPCAT3 between M1 and M2 macrophages [137]. As a result, the increased presence of M1 macrophages not only contributes to immunotherapy, but also sustains a proinflammatory tumor microenvironment that supports tumor growth at early stage [137].

Recent research has also emphasized ferroptosis as a significant form of hepatocyte death. This process can lead to inflammation, which in turn promotes the development of HCC (hepatocellular carcinoma) and compensatory cell proliferation. The activation of ATF4 has a protective effect against the progression from steatohepatitis to HCC [213]. ATF4 accomplishes this by upregulating SLC7A11, a factor that inhibits stress-related ferroptosis, thereby slowing down the onset of HCC [213]. Furthermore, experiments involving the conditional knockout of *Gpx4* in the liver have shown an acceleration of diethylnitrosamine-induced liver cancer. This acceleration is attributed to the release of HMGB1, which in turn recruits MDSC infiltration and is associated with the upregulation of CD274/PD-L1 [214].

These findings suggest that secondary tumors may arise as a potential side effect of pharmacological ferroptosis induction, underscoring the need for long-term experiments to assess this possibility in suitable animal models.

## Conclusion and perspective

Ferroptosis, characterized by iron-dependent lipid peroxidation, presents a unique avenue for targeting cancer cells with specific vulnerabilities. While recent advancements have underscored its therapeutic potential in oncology, the intricate mechanisms of ferroptosis display variability and plasticity [215, 216]. However, like a coin with two sides, we must carefully weigh the reported side effects and potential risks, as discussed in this review, against its benefits during ferroptosis therapy.

Several future directions necessitate interdisciplinary collaboration to deepen our understanding of ferroptosis mechanisms and its application in tumor therapy:

### Selective induction

Effective targeted therapy should precisely eradicate cancer cells while safeguarding normal tissues and maintaining immune surveillance. Many existing ferroptosis activators lack specificity for particular cells or tissues. While N6F11 has shown some cell-specific properties, it requires higher concentrations to achieve effectiveness compared to well-known non-selective ferroptosis activators [36]. Therefore, it is imperative to make improvements in its structure, metabolic stability, and activity.

### Molecular mechanism

The specific molecular factors that set ferroptosis apart from other forms of oxidative cell death, such as mitochondrial apoptosis, are still not well understood [21]. Additionally, it is crucial to investigate whether varying levels of oxidative stress trigger distinct types of cell death. A deeper exploration is required to comprehend the functioning of different antioxidant systems, especially when critical pathways like GPX4 fail. The ongoing challenge lies in addressing acquired resistance mechanisms in ferroptosis. Furthermore, the transition between reduced and oxidized forms of non-enzymatic antioxidants also plays a role in determining the outcome of ferroptotic cell death [217, 218].

### Biomarker identification

Monitoring ferroptosis responses necessitates diverse biomarkers to assess iron and lipid metabolism as well as associated immune responses. Several biomarkers, such as TFRC [219], ACSL4 [87], and PTGS2 (prostaglandin-endoperoxide synthase 2) [9], hyperoxidized PRDX3 [220], have been measured at the mRNA or protein levels to monitor ferroptosis responses. Blood-based biomarkers, particularly danger signals, such as HMGB1 [221], ATP [222], SQSTM1 [223], and DCN (decorin) [224], hold translational potential for clinical use. LC-MS-based redox lipidomics is invaluable for characterizing ferroptotic biomarkers *in vivo*. An outstanding question is whether an easily applicable and cost-effective biomarker or method can be developed for clinical trials.

### Benefits and side effects

Despite being designed for specific elimination of drug-resistant cancer cells, ferroptosis therapies can exhibit side effects of varying unpredictability and severity, dependent on tumor types and treatments. Vigilant monitoring and management of these side effects are critical for therapy safety and continuity. In addition to developing selectively inductive drugs, the development of targeted drug delivery systems, such as nanoparticles, is essential for enhancing therapeutic effectiveness and reducing systemic side effects.

In summary, the assessment of the advantages and limitations of ferroptosis therapies in cancer is a challenging undertaking, owing to the intricate complexities of cancer signaling, the interplay among various cell death pathways, and the dynamic stress responses associated with these treatments. Ongoing comprehensive research, advanced experimental designs, and rigorous scientific methodologies are enhancing our comprehension of these intricacies. Nevertheless, these factors remain pivotal in informing decisions regarding cancer treatment strategies.

## Data Availability

No datasets were generated or analysed during the current study.
